# Distributed apoptotic checkpoints in B cell selection, tolerance, and autoimmunity

**DOI:** 10.3389/fimmu.2026.1917733

**Published:** 2026-09-30

**Authors:** Shandré Pillay, Christian T. Mayer

**Affiliations:** Experimental Immunology Branch, Center for Cancer Research, National Cancer Institute, National Institutes of Health, Bethesda, MD, United States

**Keywords:** autoimmunity, B cell apoptosis, B cell selection, clonal deletion, germinal center, immune tolerance, plasma cells, T follicular helper cells

## Abstract

Generating a diverse B cell repertoire is essential for protective immunity, yet the required genetic alterations inevitably produce self-reactive, damaged, and non-competitive cells. To maintain immune homeostasis and ensure effective humoral immune responses, recent evidence supports the concept that a distributed network of apoptotic checkpoints operates at sequential stages of B cell development and activation. We discuss how these spatiotemporally distinct checkpoints collaborate with other tolerance mechanisms to regulate B cell selection and prevent systemic autoimmunity. Central tolerance mainly uses receptor editing to resolve high-avidity autoreactivity, while peripheral transitional B cell selection relies on B-cell activating factor and tonic B-cell receptor positive-selection signals, with cells failing to secure adequate signaling eliminated through apoptosis. Within germinal centers, apoptosis serves distinct functions in the dark and light zones, eliminating B cells damaged by somatic hypermutation and culling clones that fail to successfully acquire antigen and secure adequate T follicular helper cell help, respectively. We further discuss how extrafollicular responses provide a parallel, GC-independent pathway for autoreactive B-cell activation that is distinct from the stringent selection processes operating within germinal centers. Finally, we highlight evidence for an apoptosis-dependent process after activation that restricts the persistence of diversified autoreactive clones entering the memory B-cell and plasma-cell pools. Viewing B cell tolerance through the emerging conceptual framework of distributed apoptotic checkpoints may help explain how disruptions at distinct stages contribute to heterogeneous autoimmune pathologies and identify potential targets for therapeutic intervention.

## 1 Introduction

The adaptive humoral immune system operates under a fundamental evolutionary constraint: it must generate an immensely diverse repertoire of B cell receptors (BCRs) capable of anticipating and rapidly neutralizing emerging pathogens, while simultaneously suppressing receptors that erroneously recognize host tissues (1). This diversity is generated through variable, diversity, and joining [V(D)J] recombination during B cell development. Mediated by the recombination-activating genes RAG1 and RAG2, this combinatorial process theoretically allows for over 10¹³ unique BCR specificities (2). Upon activation by foreign antigens, this primary repertoire is subsequently diversified through somatic hypermutation (SHM) within secondary lymphoid organs (1). The molecular engine driving SHM, and the concurrent process of class switch recombination (CSR), is activation-induced cytidine deaminase (AID) (3). By deaminating cytosine to uracil within immunoglobulin (Ig) loci, AID initiates distinct DNA repair pathways that mediate SHM and CSR (4). While SHM refines antigen affinity and CSR equips antibodies with specialized effector functions (e.g., transitioning from IgM to IgG or IgA), AID-mediated diversification can also generate or enhance B-cell autoreactivity (5, 6). Furthermore, off-target AID activity and double-strand DNA breaks required for CSR can promote oncogenic chromosomal translocations and malignant transformation (7). To prevent systemic autoimmunity and lymphoid malignancy, the immune system employs multiple protective mechanisms, including programmed cell death, or apoptosis (8). Recent evidence supports the concept that B cell selection is not governed by a single dominant checkpoint or deletion event, but rather by a distributed series of checkpoints at which self-reactive, damaged, and non-competitive clones may be eliminated, consistent with the emerging conceptual framework that we refer to as the Distributed Clonal Deletion Model (9). These checkpoints operate at distinct developmental and activation stages and collectively shape the B cell repertoire while contributing to tolerance maintenance. However, the relative contributions and autoreactivity specificity of individual checkpoints remain incompletely defined. Some apoptotic events occurring at these stages may reflect broader selection or quality-control processes rather than autoreactivity-specific clonal deletion, and the extent to which individual checkpoints cooperate mechanistically to maintain tolerance remains to be established. This review examines how apoptotic checkpoints interact with other selection and tolerance mechanisms across these sequential stages of B cell development and activation to maintain humoral tolerance and prevent autoimmunity (**Figure 1**).

**Figure 1:**
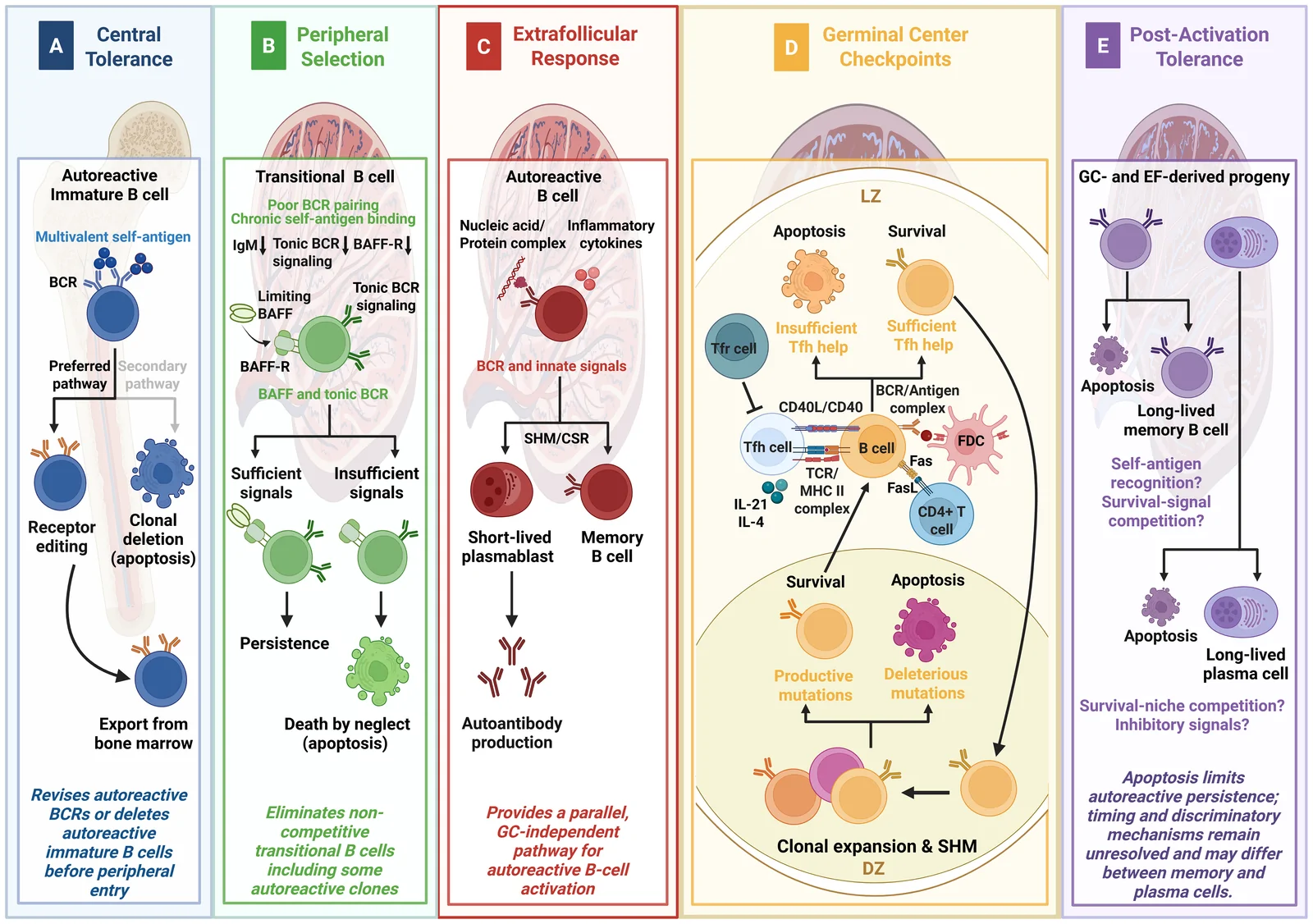
The distributed apoptotic checkpoint framework in B cell selection and humoral tolerance. **(A)** Central tolerance: Immature B cells undergo initial screening in the bone marrow. Recognition of multivalent self-antigens triggers receptor editing as the dominant tolerance mechanism; cells that fail to revise self-reactive BCR specificities may subsequently undergo apoptosis. **(B)** Peripheral selection: Transitional B-cell survival in the spleen depends on tonic BCR signaling and access to BAFF. Cells unable to secure sufficient survival signals undergo apoptosis by neglect. Autoreactive cells may additionally enter anergic states, although a fraction of low-avidity autoreactive clones persists within mature compartments. **(C)** Extrafollicular (EF) responses: A subset of activated autoreactive B cells differentiates through the EF pathway, which represents a parallel, GC-independent route rather than an apoptotic checkpoint. BCR engagement alone can induce mitochondrial dysfunction and apoptosis, whereas endosomal TLR co-stimulation provides a critical survival signal that overcomes this apoptotic constraint. Together with inflammatory cytokines, these signals promote GC-independent diversification, memory B-cell differentiation, rapid plasmablast formation, and autoantibody production outside the follicle. **(D)** GC checkpoints: GC B cells undergo iterative cycles of proliferation and somatic hypermutation in the dark zone (DZ) followed by antigen-dependent selection in the light zone (LZ). Deleterious mutations compromising BCR function are removed through intrinsic apoptosis in the DZ, whereas successful antigen acquisition from follicular dendritic cells (FDCs) and Tfh-derived signals promote survival and positive selection in the LZ. The amount and quality of Tfh-derived help determine the stringency of GC selection: competition for limiting help can indirectly constrain autoreactive clones, whereas dysregulated Tfh help may permit their persistence. Tfr cells further restrict inappropriate Tfh-mediated help and autoreactive B-cell participation and contribute to the normal termination of GC responses. Fas-mediated apoptosis provides an additional mechanism constraining inappropriate GC clones. GC apoptosis therefore provides broad quality control while also constraining autoreactive variants. **(E)** Post-activation tolerance: Apoptosis-dependent processes restrict the persistence of autoreactive antigen-experienced cells arising from both GC-dependent and GC-independent pathways. The timing and discriminatory mechanisms of this restriction remain incompletely defined and may differ between memory B-cell and plasma-cell compartments. Continued self-antigen recognition and competition for survival signals may constrain autoreactive memory B cells, whereas plasma-cell persistence may depend more strongly on access to supportive survival niches and inhibitory signals. Collectively, these selection, tolerance, and differentiation processes operate across the B-cell life cycle, with each stage shaping the repertoire substrate available to subsequent checkpoints, consistent with the emerging Distributed Clonal Deletion Model framework. Created in BioRender. Pillay, S. (2026) https://BioRender.com/gng9kt7.

## 2 Molecular regulation of B cell apoptosis

B-cell survival and death are governed by the continuous integration of external signals that activate either the intrinsic (mitochondrial) or extrinsic (death receptor) apoptotic pathways. Cellular metabolism provides an additional layer of regulation, as metabolic fitness and nutrient-sensing pathways can determine whether B cells meet the energetic requirements for development, activation, and survival; however, whether such pathways selectively discriminate autoreactive B cells and therefore constitute dedicated metabolic tolerance checkpoints remains unclear (10, 11). The intrinsic pathway acts as the default mechanism for “death by neglect” and is tightly regulated by the Bcl-2 family of proteins (12). Within this network, the pro-apoptotic BH3-only protein Bim serves as the critical sensor of antigen deprivation and insufficient survival signaling. In properly stimulated B cells, Bim is neutralized by anti-apoptotic guardians like Bcl-2 and Mcl-1 (13). When survival signals are withdrawn, anti-apoptotic proteins such as Mcl-1 decline, allowing Bim to trigger apoptosis through mitochondrial outer membrane permeabilization (MOMP) and activation of executioner caspases (14). Conversely, the extrinsic pathway operates as an active deletion switch triggered by extracellular ligands binding to transmembrane death receptors, primarily Fas (CD95). Highly expressed on activated germinal center (GC) B cells, Fas engagement by FasL (transiently expressed on helper T cells) recruits the adaptor FADD and activates caspase-8 (5, 15). Activated caspase-8 rapidly induces apoptosis of targeted B cells. In contrast, other programmed cell death pathways appear to play only minor roles in B-cell homeostasis. Genetic ablation of key mediators of pyroptosis and necroptosis produces only modest phenotypic effects (9), supporting apoptosis as the predominant mechanism regulating physiologic B-cell selection and tolerance.

Throughout this review, we distinguish apoptosis from the selection and tolerance processes in which it participates. Positive selection refers to the survival and progression of cells that obtain sufficient trophic or receptor-derived signals, whereas death by neglect refers to apoptosis resulting from insufficient survival signaling. Quality-control mechanisms eliminate damaged or functionally impaired cells without necessarily discriminating autoreactivity. Clonal deletion refers to the apoptotic elimination of individual B-cell clones; in the context of tolerance, this denotes elimination in which self-antigen recognition contributes to the death of an autoreactive clone. Here, we use “apoptotic checkpoint” to describe a developmental or activation stage at which apoptosis shapes the surviving B-cell repertoire. We use “tolerance checkpoint” only when a process contributes directly or indirectly to restricting the survival, persistence, or function of autoreactive cells; this designation does not necessarily imply a dedicated molecular sensor of autoreactivity. Accordingly, not every apoptotic event or selection process constitutes a tolerance checkpoint. The emerging Distributed Clonal Deletion Model posits that temporally distinct apoptotic checkpoints operate across developmental and activation stages and may collectively restrict the survival, diversification, and persistence of autoreactive B-cell clones (9). Importantly, apoptosis occurring at these individual checkpoints is not necessarily driven primarily by autoreactivity and may also reflect death by neglect or broader quality-control mechanisms.

## 3 Pre-activation stages: central tolerance and peripheral selection

The first mechanisms of B-cell tolerance operate during B-cell development in the specialized microenvironment of the bone marrow (**Figure 1A**), where immature B cells can encounter self-antigens in different physical forms. Recognition of multivalent, often cell-associated self-antigens arrests developmental progression and favors secondary RAG-mediated recombination to alter BCR specificity, termed receptor editing, whereas soluble self-antigens more commonly induce a functionally hyporesponsive state, termed anergy, and permit autoreactive B cells to enter the peripheral repertoire (1). Unlike thymocytes, in which secondary TCR rearrangements occur primarily before or during positive selection (16), high-avidity autoreactive immature B cells maintain or reinduce RAG expression (17), allowing receptor editing to function directly as the principal mechanism for resolving central autoreactivity. Only when receptor editing fails to eliminate self-reactivity does clonal deletion contribute (18, 19). Recent work utilizing a sensitive *in vivo* apoptosis reporter has provided a high-resolution view of apoptosis during B-cell development (20). Together with genetic studies demonstrating that inhibition of mitochondrial apoptosis fails to expand immature bone marrow B-cell populations (9), these findings suggest that physiologic clonal deletion plays a relatively limited role during bone marrow B-cell development.

IgM+ immature B cells that successfully pass central tolerance checkpoints enter the circulation as transitional B cells and migrate to the spleen. The fate of transitional B cells depends on a peripheral positive selection process that requires tonic signaling through the BCR and access to the survival cytokine B-cell activating factor (BAFF) (21–24) (**Figure 1B**). Tonic BCR signaling is critical to survival and differentiation into mature B cells and increases expression of the BAFF receptor (BAFF-R) (25–27). Cells must successfully compete for strictly limited quantities of BAFF, which activates non-canonical NF-κB signaling to sustain anti-apoptotic Bcl-2 family protein expression (28). The observation that most apoptotic transitional B cells are not autoreactive (20) suggests that peripheral selection is dominated by “death by neglect” rather than autoreactivity-specific clonal deletion, although the assays used to detect autoreactivity *in vitro* may not fully reflect self-antigen binding *in vivo*. In addition to intrinsic B-cell survival signals, studies in humans suggest that T cells and regulatory T cells also contribute to peripheral B-cell tolerance (29), although the underlying mechanisms remain incompletely understood. Nevertheless, chronic engagement with peripheral self-antigens can downregulate the IgM BCR, reduce tonic signaling, and decrease BAFF-R expression (30). Importantly, BAFF itself does not directly discriminate autoreactivity. Rather, chronic self-antigen engagement indirectly places autoreactive transitional B cells at a competitive disadvantage for limiting BAFF. Anergic B cells are excluded from B-cell follicles and prone to apoptosis (31, 32). Importantly, anergy is potentially reversible and can be overcome by appropriate antigenic or co-stimulatory signals, including particulate antigen or CD40 stimulation (33–35). BCR isotype can further modulate this peripheral selection process. Autoreactive B cells preferentially downregulate IgM while retaining IgD, which is less sensitive to endogenous antigens and thereby reduces responses to chronic self-antigen (36, 37). Recent work suggests that the long IgD hinge region raises the threshold for peripheral negative selection, allowing a broader and more polyreactive repertoire to enter the mature naïve compartment (38). In addition, chronically anergic autoreactive B cells can undergo Fas-dependent deletion following interactions with autoreactive CD4+ T cells (39), demonstrating that active apoptotic mechanisms also contribute to peripheral tolerance. While some autoreactive transitional B cells are eliminated through apoptosis and others enter short-lived anergic states, a fraction of low-avidity autoreactive cells persists within mature compartments (40). Although their persistence necessitates additional tolerance mechanisms at later developmental and activation stages, these cells may also contribute to repertoire diversity and protective immunity.

## 4 Extrafollicular responses and GC-independent autoimmunity

While highly organized follicular responses are a major source of high-affinity autoantibodies, the GC is not an absolute prerequisite for systemic autoimmunity. A significant subset of activated autoreactive B cells bypasses the GC entirely via the extrafollicular (EF) response (**Figure 1C**) (41). Within the Distributed Clonal Deletion Model, the EF response is therefore best viewed not as an apoptotic checkpoint itself, but as a parallel, GC-independent differentiation pathway available to autoreactive B cells that have escaped earlier tolerance mechanisms. Following initial activation, a subset of autoreactive B cells enters the extrafollicular pathway and migrates into EF regions, such as splenic red pulp or lymph node medullary cords, where they rapidly generate plasmablasts and other GC-independent effector populations (41, 42). The factors governing EF versus GC commitment remain incompletely understood and depend on the integration of BCR, TLR, cytokine, and T-cell-derived signals (41, 43, 44). TLR signaling can provide a critical second signal that rescues activated B cells from BCR-induced mitochondrial dysfunction and apoptosis, while inflammatory cytokines such as IL-12 and IFNγ can further bias activated B cells toward EF plasmablast differentiation (44, 45). Thus, entry into the EF pathway does not imply escape from apoptotic regulation altogether. Activated B cells remain dependent on appropriate survival signals, and the integration of BCR, innate, cytokine, and T-cell-derived signals can determine whether activated cells survive and differentiate or undergo apoptosis (41, 43–45). This pathway is particularly prominent in systemic lupus erythematosus (SLE). In SLE, circulating multivalent nuclear self-antigens, often derived from uncleared apoptotic debris, are internalized by B cells. These DNA- and RNA-containing immune complexes subsequently engage endosomal TLRs, with TLR7 playing a particularly important role in driving pathogenic autoreactive B-cell responses (43, 46, 47). This synergistic activation of the BCR and endosomal TLRs can promote robust autoreactive B-cell activation, GC-independent differentiation, and autoantibody production outside the classical GC environment (46). Although often viewed as a source of rapid plasmablast responses, EF reactions can also support AID expression, SHM, and affinity maturation, providing an additional route for the generation or enhancement of autoreactivity (41, 44). Thus, autoreactive clones may access multiple activation pathways depending on antigenic and inflammatory context.

The relative contribution of GC and EF pathways to autoimmune disease remains an area of active investigation. Nevertheless, substantial experimental evidence demonstrates that autoimmune pathology can be sustained in the absence of classical GC formation, indicating that GC-independent pathways can independently drive pathogenic autoantibody responses and disease manifestations in some settings (46, 48). Importantly, GC-independence does not imply T-cell independence. Even when classical GCs are dispensable, autoreactive EF responses often remain dependent on CD4+ T-cell-derived signals, including CD40L and IL-21, although T-cell-independent pathways have also been described (43, 49, 50). Thus, autoimmune disease can emerge through either GC-dependent or GC-independent routes. Within the Distributed Clonal Deletion Model, both pathways operate on autoreactive cells that have survived earlier checkpoints and associated selection processes, while AID-mediated diversification within these responses can also generate new autoreactive specificities. Whether autoreactive cells progress through EF or GC pathways, their capacity to drive systemic disease may be influenced by defects distributed across multiple apoptotic checkpoints and associated tolerance processes rather than by any single anatomical site of activation.

## 5 GC checkpoints: selection and apoptosis

While a subset of activated B cells differentiates through the EF pathway, others re-enter the follicle to form GCs (**Figure 1D**). These highly dynamic microanatomical structures are partitioned into a dark zone (DZ), where B cells proliferate and undergo SHM, and a light zone (LZ), where newly diversified clones compete for antigen and T follicular helper cell (Tfh)-derived survival signals (6). Tfh-mediated selection therefore acts on a repertoire already shaped by earlier central and peripheral selection processes while simultaneously evaluating clones newly generated through SHM. BCR isotype may also influence selection before GC entry, as recent evidence suggests that IgD expression on naïve B cells raises the threshold for antigen-dependent recruitment into the GC, preferentially enriching the responding population for antigen-binding, higher-affinity clones (38). The GC program, driven by Bcl-6, suppresses anti-apoptotic pathways and renders GC B cells highly dependent on external survival cues (51). In the DZ, SHM continuously generates clones carrying affinity-enhancing, neutral, and deleterious mutations. B cells that acquire damaging mutations compromising BCR function are eliminated by apoptosis, providing an essential quality-control mechanism for the mutating repertoire (52, 53). Surviving clones then migrate to the LZ, where they must acquire antigen through the BCR and present the resulting peptides to Tfh cells to receive survival and positive selection signals, primarily CD40L and IL-21 (6).

This survival signaling is highly quantitative. Antigen acquisition and the cumulative amount of Tfh help received promote pro-survival programs that oppose intrinsic apoptosis and determine the magnitude of subsequent clonal expansion upon returning to the DZ (6, 54). The availability of Tfh help therefore contributes directly to the stringency of GC selection, with limiting Tfh help promoting competition among GC B-cell clones, favoring those that acquire and present sufficient antigen (55). Conversely, dysregulated Tfh help may promote the persistence and expansion of autoreactive B-cell responses, thereby contributing to autoimmune disease (56). In addition to the quantity of Tfh help received, the quality of this help is shaped by cognate interactions and co-stimulatory and cytokine signals, including CD40L and IL-21, which influence GC B-cell proliferation, survival, and differentiation (6, 54). Clones that fail to acquire sufficient antigen, acquire mutations that reduce BCR affinity, or become poorly competitive for Tfh help are denied these rescue signals and undergo apoptosis. In addition, GC B cells express high levels of Fas, rendering them susceptible to extrinsic apoptosis following FasL engagement. Defects in either intrinsic or extrinsic apoptotic pathways can disrupt GC selection and promote autoantibody production (15, 57–60), highlighting an important role for apoptosis in humoral tolerance. Importantly, GC apoptosis appears to function as a broad quality-control mechanism rather than a dedicated autoreactivity filter, removing damaged and poorly competitive clones while simultaneously limiting the persistence of autoreactive variants (52, 61). Thus, the GC represents an important post-activation selection and quality-control stage in which intrinsic and extrinsic apoptotic pathways shape the mutating B-cell repertoire while also constraining autoreactivity.

T follicular regulatory (Tfr) cells provide an opposing regulatory influence within the follicular response. Tfr cells are a specialized population of Foxp3+ regulatory T cells that access B-cell follicles and GCs and constrain humoral responses through regulation of both Tfh and B cells. By limiting inappropriate Tfh-mediated help and B-cell activation, Tfr cells can restrict the participation and expansion of autoreactive B-cell clones within GCs (62). Tfr also contribute to terminating GC responses induced by immunization (63). Thus, the balance between Tfh-derived positive selection signals and Tfr-mediated regulation contributes to the stringency and duration of GC responses and provides an important link between T-cell regulation, B-cell survival, and maintenance of humoral tolerance.

## 6 Post-activation tolerance in memory B cells and plasma cells

Multiple lines of evidence indicate that tolerance is not completed during B-cell activation and selection, but continues during the formation and maintenance of memory B cells and plasma cells (**Figure 1E**). Self-reactive and polyreactive antibodies are markedly depleted from the human IgM+ memory B-cell compartment relative to mature naïve B cells (64), and polyreactive IgG+ memory B cells progressively decline over time in mice (65), indicating that tolerance extends into antigen-experienced B-cell populations. Direct evidence that apoptosis contributes to post-activation tolerance came from genetic mouse models that selectively inhibited MOMP after B-cell activation (61). Restricting Bcl-2 overexpression to activated AID-expressing B cells increased GC B-cell survival but did not result in the accumulation of highly autoreactive memory B cells or plasma cells. In contrast, extending apoptosis inhibition into post-activation compartments permitted the persistence of highly autoreactive memory B cells and plasma cells, including clones whose autoreactivity was generated or enhanced by SHM, resulting in sustained autoantibody production (61). These findings support an apoptosis-dependent process operating after activation that limits the persistence of autoreactive clones within long-lived memory and plasma-cell compartments.

Importantly, this process acts on cells emerging from both GC-dependent and GC-independent pathways, as both ultimately contribute to long-lived memory and plasma-cell pools. However, memory B cells and plasma cells represent biologically distinct compartments with different survival requirements and niches, and the mechanisms restricting autoreactivity within these populations should therefore not be assumed to be equivalent. Consistent with the changing survival requirements of antigen-experienced B cells, BAFF/APRIL receptor usage shifts during B-cell differentiation, with BAFF-R contributing to memory B-cell survival and TACI regulating B-cell activation and differentiation. Recent mouse studies further identify TACI as an important regulator of short- and long-lived plasma-cell maintenance, whereas BCMA appears dispensable for long-lived plasma-cell survival under the tested conditions (28, 66–68). Although these receptors clearly influence B-cell survival and humoral tolerance, their precise contribution to post-activation apoptosis-dependent tolerance remains incompletely understood. BCR isotype may provide an additional layer of regulation at this stage, as class-switched BCRs can impose distinct signaling and cell-fate programs. For example, class-switching to IgG1 favors plasma-cell over memory B-cell differentiation, while IgE BCR signaling can restrict B-cell persistence and promote plasma-cell elimination, and IgA-expressing B cells exhibit distinct requirements for BCR signaling (65, 69–72). Whether such isotype-specific mechanisms contribute more broadly to restricting the persistence of autoreactive memory B cells and plasma cells remains to be determined. The mechanisms that restrict autoreactive cells at this post-activation stage remain incompletely defined and may differ between memory B cells and plasma cells. We speculate that continued self-antigen recognition and competition for limiting survival signals could constrain autoreactive memory B cells, whereas plasma-cell tolerance may depend more strongly on competition for supportive survival niches and inhibitory signals, such as FcγRIIB engagement, which can directly promote plasma-cell apoptosis (73). Death-receptor signaling or other interactions with surrounding immune cells could provide additional mechanisms for restricting autoreactive cells within either compartment. However, autoantibody production alone is not synonymous with clinical autoimmune disease, because pathogenicity depends on factors such as antigen specificity, antibody isotype, complement activation, and the breadth of the autoreactive repertoire (9, 29, 74).

## 7 Distributed checkpoint failure and disease heterogeneity

The apoptotic checkpoints and associated selection and differentiation processes summarized in **Figure 1** operate across distinct stages of the B-cell life cycle, with each stage influencing the cellular substrate available to subsequent checkpoints. The Distributed Clonal Deletion Model (9) provides a framework for understanding how defects at different stages of B-cell development and activation may contribute to distinct disease outcomes. Because checkpoints operating at different stages act on different cellular substrates, failure of early and late checkpoints can have markedly different consequences.

Failure of early checkpoints expands the pool of autoreactive B cells available to participate in subsequent immune responses. As demonstrated by Newen et al., inhibition of MOMP beginning before B-cell activation and persisting throughout the B-cell life cycle permits broad expansion and diversification of autoreactive clones, generating a highly diverse class-switched IgG repertoire directed against systemic self-antigens and resulting in severe autoimmune disease with complement activation and tissue pathology (9). In contrast, defects restricted to activated and post-activation compartments act on autoreactive clones only after entry into the activation phase of the immune response, when earlier tolerance checkpoints have already shaped the available repertoire. Consequently, failure of late checkpoints promotes the persistence of activated autoreactive clones and their memory and plasma-cell progeny, but can produce a more restricted pattern of autoreactivity and distinct pathological outcomes (9, 61). Thus, in these experimental models, disease severity is influenced not only by the failure of individual checkpoints, but also by the developmental stage at which tolerance is disrupted.

Evidence from human autoimmune disease similarly indicates that B-cell tolerance can be disrupted at multiple stages, although the extent to which these abnormalities reflect failures of apoptosis-dependent checkpoints remains less clear. In SLE, increased frequencies of autoreactive mature naïve B cells indicate defects in early B-cell tolerance (75), while autoreactive VH4-34-expressing B cells that are normally excluded from germinal-center responses can participate in GCs in patients, consistent with defects in post-activation selection (76). Human studies of BAFF inhibition further demonstrate that B-cell survival pathways can influence autoreactive repertoire selection, with belimumab promoting counterselection of autoreactive activated B cells and plasmablasts while having limited effects on the resting naïve repertoire (77). Defects spanning multiple stages have also been observed in Sjögren disease, where autoreactive B cells accumulate within both naïve and memory compartments (78), whereas rheumatoid arthritis is associated with impaired early central and peripheral B-cell tolerance (79). Together, these observations support the existence of stage-specific abnormalities in B-cell tolerance across human autoimmune diseases. However, they do not establish that the same apoptosis-dependent mechanisms identified in experimental models underlie these abnormalities, nor that the developmental timing of checkpoint failure alone determines the clinical heterogeneity of human autoimmune disease.

Ultimately, the pathogenic consequences of checkpoint failure are influenced not simply by the number of autoreactive clones that escape deletion, but by the antigen specificity, repertoire breadth, and effector functions of the resulting autoantibodies. The Distributed Clonal Deletion Model may therefore help explain how defects arising at different stages of B-cell selection and tolerance could contribute to markedly different autoimmune phenotypes despite involving the same underlying process of autoreactive B-cell escape.

## 8 Conclusion

Available evidence supports a model in which apoptosis shapes B-cell selection and tolerance at multiple stages of development, activation, and terminal differentiation. Across these stages, apoptosis limits the survival of damaged, poorly competitive, and autoreactive clones while preserving the diversity required for effective humoral immunity. The Distributed Clonal Deletion Model provides an emerging conceptual framework that may help explain how tolerance can remain robust despite the incomplete efficiency of any individual checkpoint and why defects arising at different stages of the B-cell life cycle may contribute to distinct autoimmune phenotypes. However, the relative contributions and autoreactivity specificity of individual checkpoints remain incompletely defined. Importantly, the timing of checkpoint failure influences the autoreactive substrate available for diversification and persistence, thereby potentially shaping both the breadth of autoreactivity and the severity of resulting disease.

This framework has important therapeutic implications. Current treatments for autoimmune disease largely rely on broad immunosuppression or depletion of entire immune-cell populations, approaches that can compromise protective immunity (80). A more precise understanding of how apoptotic checkpoints are regulated may enable both selective restoration of tolerance and targeted interruption of the activation, diversification, survival, and persistence pathways that allow autoreactive clones to drive disease after checkpoint failure. Targeting pathways that regulate autoreactive clone activation, diversification, survival, and persistence, including BAFF signaling, T-cell help, Fas, and Bcl-2 family proteins, offers opportunities to preferentially constrain pathogenic autoreactive clones while preserving protective immune responses (81). Ultimately, defining how apoptotic checkpoints interact across the B-cell life cycle may provide a foundation for the development of next-generation therapies that restore immunological homeostasis with greater precision and fewer adverse effects.
